# Genomics: New Light on Alzheimer’s Disease Research

**DOI:** 10.3390/ijms19123771

**Published:** 2018-11-27

**Authors:** Yeong Ju Jung, Yoon Ha Kim, Mridula Bhalla, Sung Bae Lee, Jinsoo Seo

**Affiliations:** 1 Department of Brain & Cognitive Sciences, DGIST, Daegu 42988, Korea; jyj5028@naver.com (Y.J.J.); dbsgk1005@dgist.ac.kr (Y.H.K.); 2 Institute of Bioinformatics and Biotechnology, Savitribai Phule Pune University, Pune 411007, India; bmridula158@gmail.com

**Keywords:** alzheimer’s disease, genomics, GWAS, genetic risk factors, epigenetic modification, aging

## Abstract

Alzheimer’s disease (AD) is a progressive neurodegenerative disease that represents a major cause of death in many countries. AD is characterized by profound memory loss, disruptions in thinking and reasoning, and changes in personality and behavior followed by malfunctions in various bodily systems. Although AD was first identified over 100 years ago, and tremendous efforts have been made to cure the disease, the precise mechanisms underlying the onset of AD remain unclear. The recent development of next-generation sequencing tools and bioinformatics has enabled us to investigate the role of genetics in the pathogenesis of AD. In this review, we discuss novel discoveries in this area, including the results of genome-wide association studies (GWAS) that have implicated a number of novel genes as risk factors, as well as the identification of epigenetic regulators strongly associated with the onset and progression of AD. We also review how genetic risk factors may interact with age-associated, progressive decreases in cognitive function in patients with AD.

## 1. Introduction

Alzheimer’s disease (AD) is the most common cause of dementia, accounting for approximately 60–80% of dementia cases, followed by vascular dementia (approximately 10%), Lewy Body or Parkinson’s disease-related dementia, and alcohol-mediated dementia [1]. Mild cognitive impairment, one of the representative early symptoms of AD, makes this disease distinguishable from other types of dementia. In other cases, cognitive dysfunction occurs as a consequence of the primary pathology, such as brain atrophy due to deconstruction of the vascular system and impaired blood supply (i.e., vascular dementia) or loss of dopaminergic neurons (i.e., Parkinson’s disease) [1].

While memory loss commonly represents the pathological outcome of AD, the disease is unfortunately associated with far more devastating consequences. A recent study reported that AD is the sixth leading cause of death in the US. Moreover, between 2000 and 2015, deaths due to AD increased by 123%, aligning with increases in the proportion of older adults in the population [1]. Similar trends have been observed worldwide. In 2016, the World Alzheimer Report indicated that the number of people with dementia in most countries with welfare programs for older adults is expected to increase by 50–110% over the next 15 years [2].

Since Dr. Alois Alzheimer first described the case of his patient Auguste D. in 1906 [2], tremendous efforts have been made to develop therapeutics for AD. Senile plaques and neurofibrillary tangles are two major hallmarks observed in the brains of patients with AD. Wong et al. identified senile plaques as aggregates of β-amyloid (Aβ)—cleaved fragments of amyloid precursor protein (APP) [3]. Pedigree investigations in certain families with AD revealed that mutations in APP or presenilin (PSENs), proteins in the catalytic core of γ-secretase, cleave APP into Aβ [4,5,6]. Several studies have indicated that these mutations, which are associated with familial AD (fAD), can cause early-onset AD (before the age of 65) [7]. These findings strongly supported the idea that abnormal accumulation of Aβ initiates disease pathology (i.e., the so-called “amyloid cascade hypothesis”) [8,9]. Numerous studies have attempted to experimentally validate the pathological effects of this peptide. Such studies have reported that treatment with synthetic Aβ or Aβ isolated from postmortem samples from patients with AD induces various pathological features related to AD in neurons, including loss of synapses and neurodegeneration [10,11]. Exogenous expression of human APP and/or PSENs with disease-associated mutations in animal models leads to cognitive impairment as well as various pathological phenotypes including neuroinflammation, cellular organelle dysfunction, and impairments similar to those observed in cellular models [12,13]. Although most animal models with familial AD mutations fail to display tauopathy such as hyperphosphorylation of tau and tangle formation [12,14], these studies support the notion that increased Aβ (specifically Aβ_42_ species) production and accumulation is the primary cause of AD.

The seemingly straightforward amyloid cascade hypothesis suggests that AD progression can be prevented by reducing Aβ levels in the brain. Thus, substantial efforts have been made to develop efficient ways to reduce levels of Aβ and validate their effects on AD pathology. Immunodepletion of Aβ using anti-Aβ antibody efficiently reduces both soluble and insoluble levels of this peptide in the brains of fAD mouse models [15,16,17]. Reduced levels of Aβ in these animals correlate with the attenuation of many AD-like phenotypes, such as neuroinflammation and impaired synaptic plasticity. Moreover, several studies have reported that Aβ immunodepletion improves cognitive function in animal models of fAD, when compared to animals without Aβ immunodepletion. Various studies have also examined the effects of inhibitors of the APP-cleaving enzymes β-secretase (BACE) and γ-secretase [18,19,20]. Treatment with such inhibitors significantly reduces levels of Aβ, alleviates pathological features of AD, and recovers cognitive function in animal models of fAD. Despite such promising data, multiple candidate compounds targeting Aβ have been unsuccessful in clinical trials so far. In some cases, poor efficacy was due to poor delivery of the drug across the blood-brain barrier (BBB). However, even when appropriately delivered to the brain, many drugs that reduced Aβ levels in animal models failed to induce beneficial effects on cognitive performance when compared with placebo treatment. Recent clinical trials of monoclonal antibodies against Aβ or inhibitors of BACE have shown promise: Aducanumab (BIIB037), developed by Biogen, resulted in dose-dependent decreases in Aβ (up to 70%), suppressed the progression of cognitive decline in a phase 2 study, and has now been moved to phase 3 investigations. BAN2401, another antibody against Aβ, and Elenbecestat, a BACE inhibitor, have also shown promising results in phase 2 clinical studies.

It is important to note that most cases of AD (~95% of cases) are late-onset (LOAD), in which the onset of pathological features occurs after the age of 65 years. The causes of LOAD remain unclear [1]. Previous research has indicated that none of the mutations on genes associated with LOAD directly mediate Aβ generation [21], suggesting that factors other than Aβ play a key role in AD pathology. Moreover, the discrepancy between amyloidosis and LOAD has been presented in multiple studies, which have demonstrated that individuals carrying amyloid plaques in the brain may not experience cognitive deficits. Furthermore, one recent study suggested that up to 47% of individuals with normal cognitive function may be regarded as amyloid-positive when evaluated using positron-emission tomography (PET) [22].

During the progression of AD, tauopathy follows Aβ accumulation. Glycogen synthase kinase-3β (GSK-3β) and cyclin-dependent kinase 5 (Cdk5) are known to be primarily responsible for tau hyperphosphorylation in pathological conditions [23]. Several studies have demonstrated that Aβ induces aberrant activation of these kinases [24,25,26,27,28]. Hyperphosphorylation of tau leads to the dissociation of this protein from microtubules, which results in their disassembly. Microtubule disorganization inhibits transportation of many intracellular components, including mitochondria, likely contributing to neuronal dysfunction [29,30]. Hyperphosphorylated tau also adopts a secondary structure of paired helical filaments, which aggregate to form insoluble neurofibrillary tangles [31]. Its accumulation further contributes to the disruption of microtubule-binding activity and axonal transport. It has suggested that this causes an abnormal interaction between tau and the actin cytoskeleton, leading to tau mislocalization to dendritic spines and mediating synaptic dysfunction. Because it is almost exclusively expressed in axons, tau is often considered an axonal marker. However, hyperphosphorylation of tau causes accumulation of the protein in dendritic regions. Such mislocalization of tau also recruits its interacting proteins to uncharacteristic regions, thereby leading to abnormal cellular reactions. For example, the tau-interacting protein Fyn kinase is not normally expressed in the postsynaptic area. Once hyperphosphorylated tau is mislocalized to the dendritic synapses, Fyn kinase is also recruited to the postsynaptic area, where it abnormally phosphorylates N-methyl-d-aspartate (NMDA) receptors, resulting in excitotoxicity [32,33]. Hyperphosphorylation of tau also induces a conformational change resulting in the exposure of a sticky, hydrophobic domain, thereby leading to protein aggregation and the development of neurofibrillary tangles [31,34]. As in other forms of proteopathy, this could further trigger the aggregation of other proteins by causing defects in the cellular machinery required for protein homeostasis [35].

However, the role of tauopathy in AD pathology has been challenged. Mutations in the *MAPT* gene, which codes tau protein, are not linked to familial types of AD, suggesting that tauopathy may not be a central player in AD. Furthermore, mouse models with fAD mutations do not exhibit tau pathology in vivo [12,14]. The absence of tauopathy in fAD mouse models may be due to the short lifespan of mice, which may prevent the level of Aβ accumulation necessary to induce tauopathy. Such discrepancies may also be due to species differences between mice and humans, as the tau splicing variants expressed in mice differ from those observed in humans. Indeed, one recent study reported that tau hyperphosphorylation, abnormal tau conformational changes, and neurodegeneration were present in the brains of fAD mice with transplanted human tau, but not in control animals [36]. Studies involving human model systems including induced pluripotent stem cells (iPSCs) have also reported increased levels of phosphor-tau in neurons derived from the iPSCs of patients with fAD or sporadic AD (sAD) [37,38,39,40]. Such studies have further demonstrated that inhibiting Aβ generation leads to a reduction of tau hyperphosphorylation in these cells [40]. In this regard, although some therapeutic approaches target tau rather than Aβ, their beneficial effects in patients with AD have yet to be clinically proven. 

**Figure 1:**
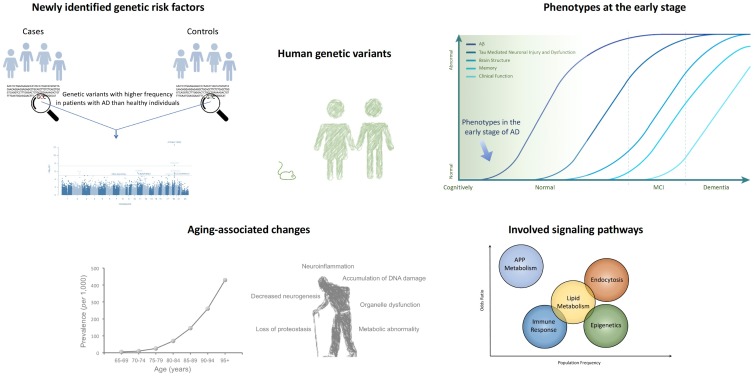
Genome-wide association studies (GWAS) have identified Alzheimer’s disease (AD)-associated genetic risk factors unique to humans, suggesting that cellular and molecular functional changes occur in the early stages of AD. Such studies have identified several signaling pathways that may be involved in AD, as well as the role of aging in pathological processes [21,41].

Despite its prevalence, there is currently no effective treatment for AD, and clinical trials of drugs targeting Aβ aggregation or tau hyperphosphorylation have been largely disappointing. Furthermore, AD diagnosis remains difficult. However, over the past century, researchers have uncovered a great deal about AD (Figure 1).

Improvements in next-generation sequencing techniques allow for whole-genome/exome sequencing and comparisons of genomic information between individuals. GWAS of patients with sAD (who usually experience late-stage onset) and healthy individuals have revealed that there are multiple single nucleotide polymorphisms (SNPs) that are highly and significantly associated with sAD [21,42]. These data suggest that, even in patients with LOAD, genetic risk factors may play a major role in disease onset and progression.

Because AD is among the major neurodegenerative diseases associated with aging [1], most research to date has focused on pathological features in aged models. For example, in studies involving fAD mice, AD-associated pathology such as Aβ accumulation, neuroinflammation, and cognitive impairment was investigated mostly in aged animals. However, recent research has identified a strong association between various SNPs and AD (even LOAD), suggesting that the early stages of AD are associated with alterations in cellular and molecular function, particularly in neural progenitors and newborn neurons.

Nonetheless, aging remains one of the strongest risk factors for AD, given that the risk of disease onset significantly increases with age [1]. In other words, even people with potential genetic risk factors for AD rarely develop the disease before 65 years of age. This suggests that AD pathogenesis may share some underlying mechanisms with aging-associated changes in the brain.

GWAS have revealed not only variations in genes associated with APP metabolism and Aβ generation, but also SNPs at genes associated with other cellular functions including the immune response, endocytosis, and cholesterol metabolism [21]. These findings, along with the recent clinical failures of Aβ-targeting candidate drugs, suggest that signaling pathways other than APP metabolism trigger the onset of AD. It is important to note that, although factors other Aβ may be associated with AD pathogenesis, these factors should result in the accumulation of Aβ—a hallmark of AD.

Lastly, most research to date has relied on non-human animal model systems to investigate AD. Indeed, because obtaining live human brain samples is challenging, postmortem samples from patients with AD and age-matched controls are often used for biochemical studies. However, the quality of these samples depends on the time of collection and sample processing after death, and discrepancies among postmortem studies are abundant. Utilizing embryonic stem cells to generate human brain cell types raises ethical issues. In this regard, it is not surprising that animal models have been used to investigate AD. In these animals, various pathological phenotypes related to AD, including cognitive dysfunction, have been observed in vivo. However, unfortunately, species differences exist between human and non-human animal models. Specifically, the genomic sequences of most genes known to be associated with AD are not 100% identical between human and animal models. Furthermore, animal models do not endogenously carry the SNPs frequently found in patients with AD. Therefore, human genes containing the appropriate variants are often overexpressed in animal models in order to study the role of disease-associated SNPs [43]. Mouse models are easy to handle and manipulate, and they have a relatively short lifespan compared to non-human primate models such as chimpanzees and monkeys. Thus, transgenic mice overexpressing APP or PSENs with fAD mutations have been widely utilized in AD research, given that they nicely recapitulate most AD-related pathologies including Aβ plaque formation and cognitive impairment [43,44].

Unfortunately, as previously mentioned, fAD mutation fails to cause tauopathy. Lack of tauopathy in the mouse brain may be due to the absence of an interaction between introduced humanized fAD gene/protein and murine proteins initiating tauopathy or tau itself. Based on this idea, researchers have additionally introduced humanized tau into the brains of fAD mice, although this has resulted in relatively minor effects on tauopathy. It is possible that, even though tau has been humanized, effector proteins interacting with Aβ and initiating tau hyperphosphorylation are still not humanized in these fAD mouse models. In addition, overexpression of exogenous genes may cause non-specific pathological phenotypes such as endoplasmic reticulum stress in animal models, which hinders researchers from distinguishing pathological roles of disease-associated mutation from the non-specific effects caused by overexpression [45]. These weaknesses of animal models are often cited as the reason why many candidate drugs that have shown beneficial effects on AD pathology in animal models have failed in clinical trials. Thus, to avoid the tremendous expense associated with such failures, it is necessary to develop human model systems in order to elucidate the cellular and molecular functions of genetic risk factors and validate the beneficial effects of candidate drugs on AD pathology.

In this review, we further discuss how GWAS have expanded our knowledge of AD, as well as the directions of future AD research.

## 2. GWAS Shed New Light on AD

To date, AD research has mainly focused on early-onset familial cases driven by mutations on *APP* or *PSEN*. Although they only comprise approximately 5% of AD cases, the cause of pathology seems to be quite straightforward, as mutations in these genes are well known to induce either increases in total Aβ production or increases in the ratio of toxic Aβ species, eventually resulting in neurodegeneration [46]. In contrast, the causes of pathology in late-onset cases remains to be determined. As previously mentioned, recent technological advancements in genomics have led to the identification of several potential genetic risk factors for LOAD [47]. As most cases of AD are late-onset, genetic risk factors for AD etiology in these cases are of high value [48].

In GWAS, researchers attempt to identify SNPs associated with AD by statistically analyzing the frequency of variants in the genome of the disease group in relation to the control group. Although the reported genetic risk factors for AD vary among studies depending on sample size, location of collection, and methods for data analysis, data from recent studies involving thousands of patients have identified associations for *APOE*, *CLU*, *CR1*, *BIN1*, *PICALM*, *CD33*, *ABCA7*, *CD2AP*, *EPHA1*, *MS4*, and *TREM2* [42,49,50,51]. Interestingly, genetic risk factors revealed through GWAS are located on or near the genes involved in various cellular signaling pathways including cholesterol metabolism, immune response, and endocytosis. These data highlight the potential contribution of these pathways to AD pathology [21].

Among the genetic risk factors uncovered, the ε4 allele of *APOE* is one of the strongest SNPs associated with AD [52,53]. Although ApoE is mainly produced and secreted by astrocytes in the brain, recent studies have reported substantial expression of ApoE in microglia [54,55,56,57]. Moreover, ApoE synthesis is known to increase during injury and neurodegeneration [55]. Given its major role in transporting lipids, (including cholesterol) across cells [58], studies have suggested that ApoE regulates levels of Aβ via physical interactions [59,60]. Human ApoE is encoded by the *APOE* gene located on chromosome 19q 13.2 and exists as three isoforms (ε2, ε3 and ε4). ApoE3, the most common isoform, contains cysteine (Cys), whereas ApoE4 contains arginine (Arg), at position 112 near the receptor-binding region (residues 136–150) of the N-terminal domain. The difference between ApoE3 and ApoE2 is observed at position 158, where ApoE2 encodes Cys instead of Arg [60,61]. Such single amino acid substitutions are suggested to affect the structure of the gene product and result in differences in their binding ability to lipids, receptors, and Aβ. ApoE4 is the isoform associated with AD. In autopsy-based studies, the risk of AD is increased for Caucasian patients with one or two copies of the ε4 allele, when compared to those with two copies of the ε3 allele: ε2/ε4 (odds ratio: OR = 2.6), ε3/ε4 (OR = 3.2), and ε4/ε4 (OR = 14.9). The *APOE4* allele for AD appears to be more frequent among Japanese patients than among Caucasian, African-American, or Hispanic patients, suggesting that ORs may vary based on ethnicity [60,62]. The underlying mechanisms of ApoE4-associated AD pathogenesis have been extensively studied since its strong association with AD was first reported [52,63]. The isoforms of ApoE appear to influence the degree of Aβ clearance differently, in the following order: ApoE4 < ApoE3 < ApoE2, possibly due to differences in binding affinity for Aβ, as described above. Several researchers have further reported that ApoE4 may contribute to various functional abnormalities related to AD in multiple ways by causing neurotoxicity; inducing synaptic dysfunction; and exacerbating neuroinflammation, mitochondrial dysfunction, and cerebrovascular defects [64].

**Figure 2:**
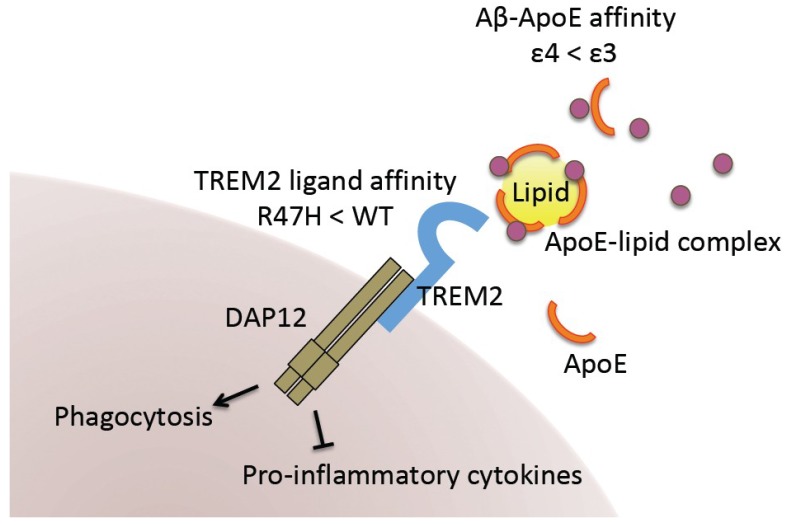
Impact of APOE4 and TREM2 R47H variants on AD pathology. Lipidated ApoE binds Aβ for its internalization or transport to other cell types. ApoE4 exhibits lower Aβ-binding affinity than ApoE3. TREM2 in microglia mediates ApoE-driven Aβ clearance. It is also required for microglial phagocytosis and inhibition of the immune response. The genetic variant on this gene (*TREM2* R47H) has been shown to exhibit lower ligand-binding affinity.

A rare variant of the *TREM2* gene (R47H, rs75932628) is another strong genetic risk factor for AD, leading to a two-fold increase in the risk of its occurrence [21,51,65]. The *TREM2* gene is located on chromosome 6p21.1 and encodes a transmembrane receptor [66]. In the brain, TREM2 is primarily expressed in microglia, the cell type that mediates synapse refinement and maintains the microenvironment for homeostasis in brain. TREM2 is involved in the refinement of synapses involved in memory formation. For example, in the developing brain, TREM2 is crucial for microglia to prune supernumerary synapses [67]. TREM2 has also been reported to mediate multiple functions of microglia such as phagocytosis, inhibition of inflammatory signaling, and promotion of cell survival [68]. TREM2 is reported to be involved in signaling pathways involving binding ligands such as anionic bacterial production and phospholipids [69]. For instance, TREM2 activation in microglia upon ligand binding induces a signaling cascade including DAP12 phosphorylation, resulting in increased phagocytosis and decreased pro-inflammation [70,71,72]. Therefore, to fully understand the role of the TREM2 variant in AD pathogenesis, researchers should aim to elucidate how this variant alters microglial function. TREM2 R47H has been reported to decrease binding affinity of TREM2 for lipid ligands and the clearance of lipoprotein particles. Although the mechanisms of lipid-Aβ complex clearance in the brain are still under investigation, it is possible that TREM2 R47H contributes to Aβ accumulation by attenuating microglial-mediated Aβ clearance (Figure 2). Nonetheless, it remains to be determined whether TREM2 R47H leads to loss-of-function or gain-of-function with regard to TREM2.

Advancements in genomics and GWAS, in particular, have helped to elucidate the genetic variants associated with AD. Given the identification of genetic risk factors for AD, many recent studies have focused on the relationships between these variants and AD, especially the mechanisms underlying pathogenesis, using various model systems including human iPSCs. Previous studies have identified some genetic variants associated with insulin signaling and type II diabetes, suggesting that further studies may help to uncover the relationship between insulin signaling and AD pathogenesis [73]. As mentioned above, such studies have also demonstrated that many of these genetic risk factors are mainly expressed in microglia, including *APOE* and *TREM2.* Such findings support the notion that alterations in microglia play a role in AD progression. Nevertheless, given that Aβ is a major hallmark of AD, future research should focus on how these cellular signaling pathways are associated with factors affecting the production or clearance of Aβ. This approach will establish a foothold for the development of more effective treatments for AD.

## 3. Epigenetic Modification: Changes in Gene Expression without Genomic Alteration

Because many studies have reported that AD is associated with changes in the expression of numerous genes, such transcriptomic changes are expected to be closely connected to AD pathology [74]. In addition, since AD is mostly late-onset and the frequency of familial cases is low [75], transcriptional changes caused by epigenetic changes that do not affect the DNA sequence may have a greater impact on onset than direct genetic mutations. LOAD is thought to be a multifactorial disease that is affected by both genetic mutation and epigenetic changes [76,77,78]. Notably, epigenetic changes are widely used as a biomarker of memory-related function, impairments in which represent the main symptom of AD [79]. However, postmortem studies of the brains of patients with AD cannot be used to draw causal inferences regarding the role of epigenetic modification in AD pathogenesis. Nonetheless, these data clearly indicate a correlation between epigenetic changes and AD.

**Figure 3:**
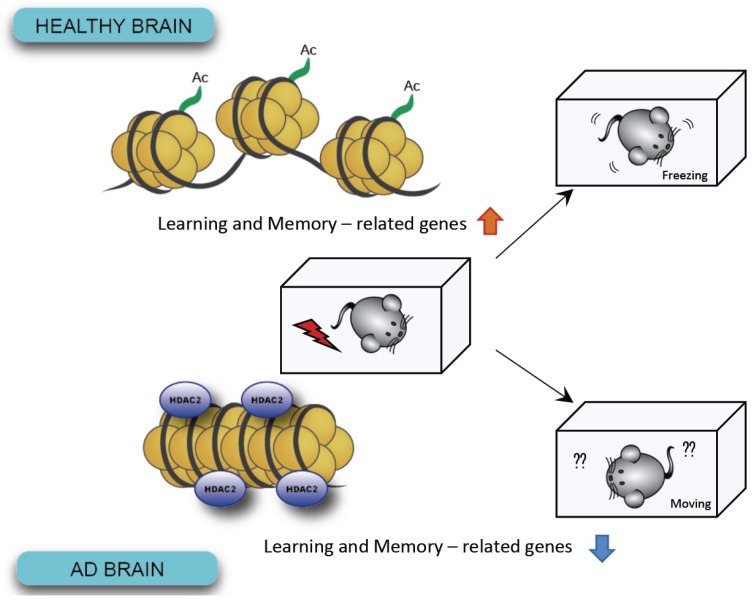
In AD brains, increased levels of HDAC2 have been shown to lead to defective chromosomes and reduced expression of genes associated with learning and memory.

Histone acetylation by histone acetyltransferases (HATs) and deacetylation by histone deacetylases (HDACs), which remove acetyl groups from an ε-N-acetyl lysine on a histone, are the most ubiquitous modifications that induce increases or decreases in gene transcription [80]. As previously described, loss of memory in AD contexts may be closely related to such epigenetic changes [81]. For example, levels of histone acetylation at H3K18/K23 in the temporal lobe are lower in patients with AD than in age-matched controls [82]. Among several epigenetic changes, histone deacetylation by HDAC2 (belonging to HDAC class I) in the hippocampus is known to have a detrimental effect on memory function. Previous studies have reported that mice overexpressing HDAC2 exhibit impaired hippocampal-dependent memory formation and reduced synapse formation [83]. Moreover, HDAC2 levels are upregulated in the hippocampal CA1 and prefrontal cortex of AD model mice, while chromatin immunoprecipitation (ChiP) assays have indicated that HDAC2 is specifically enriched in genes involved in learning and memory or synaptic plasticity [84]. Additional studies have indicated that reducing HDAC2 levels attenuates pathological features in AD model mice [85]. These data indicate that HDAC2 may mediate cognitive impairment in AD (Figure 3). Current studies aim to investigate the association between HDAC2 and memory restoration. However, Pascoal and colleagues of McGill University recently reported that while investigating HDAC expression patterns using the HDAC ligand Martinostat, class I HDAC levels are reduced in the AD brain, in contrast to expectations. Because the tracer used in this study does not specifically bind each member of the HDAC class I (HDAC1, 2, 3) [86,87], further studies are required to determine precisely which HDAC is affected in the brains of patients with AD. Each HDAC appears to exert different functions, and one previous study reported that HDAC1 exerts neuroprotective effects in rodent models [88,89]. Therefore, it may be necessary to develop therapeutic agents with more specific targets.

In addition to memory-related genes, previous studies have reported that genes involved in the immune response undergo epigenetic changes in the progression of AD [90]. Because toxic proteins such as Aβ or tau are removed by the immune system in the brain [91,92], dysfunction in the clearance pathways leads to accumulation of the peptide and subsequent neuronal toxicity [93]. Specifically, the gene regions relevant to the immune response exhibit increased enhancer signatures, such as enrichment of H3K4me3 in promoters and H3K27ac in enhancers, in both patients with AD and mouse models [90]. Altered expression of immune response genes has been consistently associated with AD [94], while additional research has indicated that activated immune cells can improve cognitive and memory abilities in patients with AD [92].

Since most cases of AD are late-onset, aging is an indisputable risk factor for the disease. Aging is also associated with epigenetic changes [95]. However, it remains controversial whether the epigenetic alterations observed in the AD brain are similar to aging-dependent epigenetic changes. Low levels of histone acetylation in patients with AD appear to correlate with age-associated decreases in histone acetylation, indicating that an epigenetic change induced by aging may be further accelerated in AD [96,97]. However, one recent study profiled the genome-wide enrichment of H4K16ac in the lateral lobe of postmortem brain tissues from young and older patients with normal cognitive function, and from patients with AD. This study revealed that, although both normal aging and AD altered the distribution of H4K16ac, these processes did not appear to be associated with one another. Significant decreases in H4K16ac were observed in the AD group, whereas H4K16ac was enriched in normal aging samples [98]. Studies investigating the relationship between epigenetic changes driven by aging and AD are still in progress.

Although it remains unclear whether epigenetic changes are the primary cause of AD or result from the progression of the disease, studies have demonstrated that epigenetic manipulation sufficiently induces AD-associated pathology. Moreover, researchers have suggested that inducing epigenetic changes can mitigate AD symptoms in animal models [99]. In this regard, drugs correcting epigenetic alterations may aid in the treatment of AD pathology, including memory loss.

## 4. Aging is still One of the Greatest Risk Factors for AD

Although recent genomic studies have revealed the contributions of genetic risk factors to AD onset, pathological features of the disease tend to emerge with age [1]. Such findings clearly indicate that aging is one of the strongest risk factors for AD. During aging, our body undergoes various changes such as accumulation of DNA damage, neuroinflammation, loss of proteostasis, decreased neurogenesis, and the development of metabolic abnormalities [100]. Such changes have been shown to lead to decreased homeostasis, synaptic dysfunction, and cognitive impairment. These age-associated changes, in combination with genetic risk factors, may accelerate the progression of AD.

Cognitive decline occurs with age even in the absence of AD, although this age-associated change appears subtle when compared to the changes accompanying AD pathology. Several recent studies have begun to uncover the specific roles of each brain region. These studies have revealed that the prefrontal cortex and hippocampus are strongly associated with cognitive functions such as executive performance and memory formation. Based on the results of initial studies involving humans and non-human primates (NHPs), neuronal loss has long been suspected as a primary cause of age-associated decreases in cognitive function [101,102,103]. However, more recent studies utilizing advanced methods have proposed that there is minimal, if any, loss of neurons in the brain during normal aging, except for that in limited brain regions such as the cerebellum and substantia nigra [104,105,106,107,108]. Moreover, these studies have argued that such losses would not account for the defects in memory and aging observed in older adults. To address this existing discrepancy, further studies have suggested that age-associated cognitive impairment is rather caused by age-dependent loss of synapses. This “synaptic aging” includes both the structural collapse of dendritic branches and alterations in synaptic components such as the glutamate receptors required for cellular communication and synaptic plasticity [109,110]. Excitatory synapses are exclusively formed at dendritic spines, the small membranous protrusions of dendrites that receive excitatory synaptic inputs. Multiple studies have indicated that loss of spines occurs with age [111,112], with especially pronounced losses in the thin spines associated with learning [113]. Thus, in general, age-dependent dendritic atrophy and loss of spines most likely lead to decreased synaptic transmission and synaptic plasticity. Such reductions in synaptic strength have been shown to recruit microglia to engulf and eliminate synapses (i.e., synaptic pruning), further facilitating synapse loss [114,115,116].

As in the aging brain, synaptic loss and reductions in the number of spines are the major pathological features of AD. Previous studies have reported that treatment with Aβ oligomers is associated with profound loss of dendritic spines and synapses [10,11]. Transcriptome analyses comparing results obtained from AD brain samples and their controls revealed that AD is associated with reductions in learning and memory-related transcription [90,117]. Such impairments in synaptic strength due to factors associated with AD may initiate synaptic loss, potentially forming a feedback loop with age-mediated synaptic weakening (synaptic aging), thereby resulting in further decreases in synaptic efficacy. Synaptic weakening in the AD brain may also be initiated by aberrant synaptic pruning activity. One recent study demonstrated that up-regulation of C1q (an “eat-me” signal) in the AD brain can be attributed to increased synapse elimination by microglia [118]. In addition to reductions in basal levels of synapses, learning-induced formation of de novo spines is impaired in the brains of fAD mouse models, thereby resulting in cognitive dysfunction [119].

As mentioned above, a wealth of literature supports the notion that Aβ aggregates per se are toxic to local neurons, and that their presence results in widespread synapse dysfunction, loss, and eventual neuronal death. While Aβ peptides directly induce cellular toxicity via multiple mechanisms including aberrant activation of NMDA receptors, microgliosis, and reactive oxygen species (ROS) production, they also exert a deleterious effect on neurons via indirect mechanisms, such as simultaneously inhibiting the proteasomal degradation of damaged proteins and inducing the unfolded protein response. It is important to note that defects in the unfolded protein response, the ubiquitin-proteasome pathway, and autophagy are also facilitated with age. This provides an excellent example of how amyloidosis and aging act synergistically to provoke AD pathology in the aged brain. In such cases, impaired proteostasis is followed by endoplasmic reticulum stress, mitochondrial dysfunction, and ultimately reductions in protein synthesis to a level unable to support synapse function and neuronal survival.

## 5. Conclusions

Although the development of drugs targeting Aβ in the last century has been unfruitful, GWAS have substantially increased our understanding of the causes, implications, and potential treatment strategies for AD. Genomic studies have shed new light on the mechanisms underlying the onset and progression of AD. However, it remains unclear whether amyloidosis initiates AD pathology, even in sporadic forms of AD. Newly identified genetic variants from patients with LOAD suggest that signaling pathways associated with neuroinflammation (rather than Aβ metabolism) can trigger disease onset in such cases. Nonetheless, further studies are required to determine how these genetic variants interact with aging to induce AD pathology. Epigenetic modifications can transform our genome (without genetic risk factors) to be more prone to AD. Because environmental factors are known to affect the function of epigenetic regulators, this may be an example of how aging interacts with our genome to influence AD development.

Technical advancements will further expand our knowledge of the contribution of genomics to AD pathology—a field so vast that the issue remains to be fully addressed. For example, several studies have reported that the risk of AD increases if an individual’s mother has been diagnosed with the disease. This finding, coupled with the strong association between mitochondrial dysfunction and AD pathogenesis, has increased focus on the role of mitochondrial DNA (mtDNA) in AD. Recent studies have also suggested a potential relationship between genetic variants in mtDNA and AD, although the sample sizes (up to approximately 3000 samples) are currently insufficient for drawing a solid conclusion when compared to those from nuclear genome studies [120,121].

Genomic studies have led AD researchers to extend their scope beyond the role of Aβ, which has been the primary target for well over a decade. Hopefully, understanding the genomics of AD will lead to the identification of novel pathways for intervention.

## Abbreviations

MAPT	Microtubule associated protein tau
APOE	Apolipoprotein E
CLU	Clusterin
CR1	Complement C3b/C4b receptor 1
BIN1	Bridging integrator 1
PICALM	Phosphatidylinositol binding clathrin assembly protein
CD33	CD33 molecule
ABCA7	ATP binding cassette subfamily A member 7
CD2AP	CD2 associated protein
EPHA1	EPH receptor A1
MS4	Membrane spanning 4-domains
TREM2	Triggering receptor expressed on myeloid cells 2
H3K18/23	Histone H3 lysine 18/23
H3K4me3	Tri-methylation on histone H3 lysine 4
H3K27ac	Acetylation on histone H3 lysine 27
H4K16ac	Acetylation on histone H4 lysine 16
NMDA	N-methyl-d-aspartate

## References

[1] Alzheimer’s Association (2018). 2018 Alzheimer’s disease facts and figures. Alzheimer’s & Dementia.

[2] Prince M.J., Comas-Herrera A., Knapp M., Guerchet M.M., Karagiannidou M. (2016). World Alzheimer Report 2016—Improving Healthcare for People Living with Dementia: Coverage, Quality and Costs Now and in the Future.

[3] Maurer K., Volk S., Gerbaldo H. (1997). Auguste D and Alzheimer’s disease. Lancet.

[4] Glenner G.G., Wong C.W. (1984). Alzheimer’s disease: initial report of the purification and characterization of a novel cerebrovascular amyloid protein. Biochem. Biophys. Res. Commun..

[5] Sherrington R., Rogaev E.I., Liang Y., Rogaeva E.A., Levesque G., Ikeda M., Chi H., Lin C., Li G., Holman K. (1995). Cloning of a gene bearing missense mutations in early-onset familial Alzheimer’s disease. Nature.

[6] Mullan M., Crawford F., Axelman K., Houlden H., Lilius L., Winblad B., Lannfelt L. (1992). A pathogenic mutation for probable Alzheimer’s disease in the APP gene at the N-terminus of beta-amyloid. Nat. Genet..

[7] St George-Hyslop P.H., Tanzi R.E., Polinsky R.J., Haines J.L., Nee L., Watkins P.C., Myers R.H., Feldman R.G., Pollen D., Drachman D. (1987). The genetic defect causing familial Alzheimer’s disease maps on chromosome 21. Science.

[8] Bertram L., Tanzi R.E. (2008). Thirty years of Alzheimer’s disease genetics: The implications of systematic meta-analyses. Nat. Rev. Neurosci..

[9] Selkoe D.J. (1991). The molecular pathology of Alzheimer’s disease. Neuron.

[10] Hardy J.A., Higgins G.A. (1992). Alzheimer’s disease: The amyloid cascade hypothesis. Science.

[11] Walsh D.M., Klyubin I., Fadeeva J.V., Cullen W.K., Anwyl R., Wolfe M.S., Rowan M.J., Selkoe D.J. (2002). Naturally secreted oligomers of amyloid beta protein potently inhibit hippocampal long-term potentiation in vivo. Nature.

[12] Lesne S., Koh M.T., Kotilinek L., Kayed R., Glabe C.G., Yang A., Gallagher M., Ashe K.H. (2006). A specific amyloid-beta protein assembly in the brain impairs memory. Nature.

[13] Oakley H., Cole S.L., Logan S., Maus E., Shao P., Craft J., Guillozet-Bongaarts A., Ohno M., Disterhoft J., Van Eldik L. (2006). Intraneuronal beta-amyloid aggregates, neurodegeneration, and neuron loss in transgenic mice with five familial Alzheimer’s disease mutations: Potential factors in amyloid plaque formation. J. Neurosci..

[14] Radde R., Bolmont T., Kaeser S.A., Coomaraswamy J., Lindau D., Stoltze L., Calhoun M.E., Jäggi F., Wolburg H., Gengler S. (2006). Abeta42-driven cerebral amyloidosis in transgenic mice reveals early and robust pathology. EMBO Rep..

[15] Van Leuven F. (2000). Single and multiple transgenic mice as models for Alzheimer’s disease. Prog. Neurobiol..

[16] Lee E.B., Leng L.Z., Zhang B., Kwong L., Trojanowski J.Q., Abel T., Lee V.M.-Y. (2006). Targeting amyloid-beta peptide (Abeta) oligomers by passive immunization with a conformation-selective monoclonal antibody improves learning and memory in Abeta precursor protein (APP) transgenic mice. J. Biol. Chem..

[17] Sevigny J., Chiao P., Bussière T., Weinreb P.H., Williams L., Maier M., Dunstan R., Salloway S., Chen T., Ling Y. (2016). The antibody aducanumab reduces Aβ plaques in Alzheimer’s disease. Nature.

[18] Xing H.-Y., Li B., Peng D., Wang C.-Y., Wang G.-Y., Li P., Le Y.-Y., Wang J.M., Ye G., Chen J.-H. (2017). A novel monoclonal antibody against the N-terminus of Aβ1-42 reduces plaques and improves cognition in a mouse model of Alzheimer’s disease. PLoS ONE.

[19] Coimbra J.R.M., Marques D.F.F., Baptista S.J., Pereira C.M.F., Moreira P.I., Dinis T.C.P., Santos A.E., Salvador J.A.R. (2018). Highlights in BACE1 Inhibitors for Alzheimer’s Disease Treatment. Front Chem..

[20] Ridler C. (2018). Alzheimer disease: BACE1 inhibitors block new Aβ plaque formation. Nat. Rev. Neurol..

[21] Wolfe M.S. (2012). γ-Secretase inhibitors and modulators for Alzheimer’s disease. J. Neurochem..

[22] Karch C.M., Goate A.M. (2015). Alzheimer’s disease risk genes and mechanisms of disease pathogenesis. Biol. Psychiatry.

[23] Plattner F., Angelo M., Giese K.P. (2006). The roles of cyclin-dependent kinase 5 and glycogen synthase kinase 3 in tau hyperphosphorylation. J. Biol. Chem..

[24] Llorens-Martín M., Jurado J., Hernández F., Avila J. (2014). GSK-3β, a pivotal kinase in Alzheimer disease. Front Mol. Neurosci..

[25] Takashima A. (2006). GSK-3 is essential in the pathogenesis of Alzheimer’s disease. J. Alzheimers Dis..

[26] Patrick G.N., Zukerberg L., Nikolic M., de la Monte S., Dikkes P., Tsai L.H. (1999). Conversion of p35 to p25 deregulates Cdk5 activity and promotes neurodegeneration. Nature.

[27] Seo J., Giusti-Rodríguez P., Zhou Y., Rudenko A., Cho S., Ota K.T., Park C., Patzke H., Madabhushi R., Pan L. (2014). Activity-dependent p25 generation regulates synaptic plasticity and Aβ-induced cognitive impairment. Cell.

[28] Seo J., Kritskiy O., Watson L.A., Barker S.J., Dey D., Raja W.K., Lin Y.-T., Ko T., Cho S., Penney J. (2017). Inhibition of p25/Cdk5 Attenuates Tauopathy in Mouse and iPSC Models of Frontotemporal Dementia. J. Neurosci..

[29] Mazanetz M.P., Fischer P.M. (2007). Untangling tau hyperphosphorylation in drug design for neurodegenerative diseases. Nat. Rev. Drug Discov..

[30] Kondadi A.K., Wang S., Montagner S., Kladt N., Korwitz A., Martinelli P., Herholz D., Baker M.J., Schauss A.C., Langer T. (2014). Loss of the m-AAA protease subunit AFG₃L₂ causes mitochondrial transport defects and tau hyperphosphorylation. EMBO J..

[31] Kolarova M., García-Sierra F., Bartos A., Ricny J., Ripova D. (2012). Structure and pathology of tau protein in Alzheimer disease. Int. J. Alzheimers Dis..

[32] Ittner L.M., Ke Y.D., Delerue F., Bi M., Gladbach A., van Eersel J., Wölfing H., Chieng B.C., Christie M.J., Napier I.A. (2010). Dendritic function of tau mediates amyloid-beta toxicity in Alzheimer’s disease mouse models. Cell.

[33] Ittner A., Ittner L.M. (2018). Dendritic Tau in Alzheimer’s Disease. Neuron.

[34] Querfurth H.W., LaFerla F.M. (2010). Alzheimer’s disease. N. Engl. J. Med..

[35] Jucker M., Walker L.C. (2011). Pathogenic protein seeding in Alzheimer disease and other neurodegenerative disorders. Ann. Neurol..

[36] Espuny-Camacho I., Arranz A.M., Fiers M., Snellinx A., Ando K., Munck S., Bonnefont J., Lambot L., Corthout N., Omodho L. (2017). Hallmarks of Alzheimer’s Disease in Stem-Cell-Derived Human Neurons Transplanted into Mouse Brain. Neuron.

[37] Israel M.A., Yuan S.H., Bardy C., Reyna S.M., Mu Y., Herrera C., Hefferan M.P., Van Gorp S., Nazor K.L., Boscolo F.S. (2012). Probing sporadic and familial Alzheimer’s disease using induced pluripotent stem cells. Nature.

[38] Muratore C.R., Rice H.C., Srikanth P., Callahan D.G., Shin T., Benjamin L.N.P., Walsh D.M., Selkoe D.J., Young-Pearse T.L. (2014). The familial Alzheimer’s disease APPV717I mutation alters APP processing and Tau expression in iPSC-derived neurons. Hum. Mol. Genet..

[39] Wang C., Najm R., Xu Q., Jeong D.-E., Walker D., Balestra M.E., Yoon S.Y., Yuan H., Li G., Miller Z.A. (2018). Gain of toxic apolipoprotein E4 effects in human iPSC-derived neurons is ameliorated by a small-molecule structure corrector. Nat. Med..

[40] Raja W.K., Mungenast A.E., Lin Y.-T., Ko T., Abdurrob F., Seo J., Tsai L.-H. (2016). Self-Organizing 3D Human Neural Tissue Derived from Induced Pluripotent Stem Cells Recapitulate Alzheimer’s Disease Phenotypes. PLoS ONE.

[41] Jack C.R., Knopman D.S., Jagust W.J., Shaw L.M., Aisen P.S., Weiner M.W., Petersen R.C., Trojanowski J.Q. (2010). Hypothetical model of dynamic biomarkers of the Alzheimer’s pathological cascade. Lancet. Neurol..

[42] Lambert J.C., Ibrahim-Verbaas C.A., Harold D., Naj A.C., Sims R., Bellenguez C., DeStafano A.L., Bis J.C., Beecham G.W., Grenier-Boley B. (2013). Meta-analysis of 74,046 individuals identifies 11 new susceptibility loci for Alzheimer’s disease. Nat. Genet..

[43] Onos K.D., Sukoff Rizzo S.J., Howell G.R., Sasner M. (2016). Toward more predictive genetic mouse models of Alzheimer’s disease. Brain Res. Bull..

[44] Götz J., Ittner L.M. (2008). Animal models of Alzheimer’s disease and frontotemporal dementia. Nat. Rev. Neurosci..

[45] Sasaguri H., Nilsson P., Hashimoto S., Nagata K., Saito T., De Strooper B., Hardy J., Vassar R., Winblad B., Saido T.C. (2017). APP mouse models for Alzheimer’s disease preclinical studies. EMBO J..

[46] Hardy J., Selkoe D.J. (2002). The amyloid hypothesis of Alzheimer’s disease: Progress and problems on the road to therapeutics. Science.

[47] Ku C.S., Loy E.Y., Pawitan Y., Chia K.S. (2010). The pursuit of genome-wide association studies: Where are we now?. J. Hum. Genet..

[48] Bagyinszky E., Youn Y.C., An S.S.A., Kim S. (2014). The genetics of Alzheimer’s disease. Clin. Interv. Aging.

[49] Harold D., Abraham R., Hollingworth P., Sims R., Gerrish A., Hamshere M.L., Pahwa J.S., Moskvina V., Dowzell K., Williams A. (2009). Genome-wide association study identifies variants at CLU and PICALM associated with Alzheimer’s disease. Nat. Genet..

[50] Naj A.C., Jun G., Beecham G.W., Wang L.-S., Vardarajan B.N., Buros J., Gallins P.J., Buxbaum J.D., Jarvik G.P., Crane P.K. (2011). Common variants at MS4A4/MS4A6E, CD2AP, CD33 and EPHA1 are associated with late-onset Alzheimer’s disease. Nat. Genet..

[51] Guerreiro R., Wojtas A., Brás J., Carrasquillo M., Rogaeva E., Majounie E., Cruchaga C., Sassi C., Kauwe J.S.K., Younkin S. (2013). TREM2 variants in Alzheimer’s disease. N. Engl. J. Med..

[52] Corder E.H., Saunders A.M., Strittmatter W.J., Schmechel D.E., Gaskell P.C., Small G.W., Roses A.D., Haines J.L., Pericak-Vance M.A. (1993). Gene dose of apolipoprotein E type 4 allele and the risk of Alzheimer’s disease in late onset families. Science.

[53] Bu G. (2009). Apolipoprotein E and its receptors in Alzheimer’s disease: Pathways, pathogenesis and therapy. Nat. Rev. Neurosci..

[54] Pimenova A.A., Marcora E., Goate A.M. (2017). A Tale of Two Genes: Microglial Apoe and Trem2. Immunity.

[55] Ignatius M.J., Gebicke-Härter P.J., Skene J.H., Schilling J.W., Weisgraber K.H., Mahley R.W., Shooter E.M. (1986). Expression of apolipoprotein E during nerve degeneration and regeneration. Proc. Natl. Acad. Sci. USA.

[56] Boyles J.K., Pitas R.E., Wilson E., Mahley R.W., Taylor J.M. (1985). Apolipoprotein E associated with astrocytic glia of the central nervous system and with nonmyelinating glia of the peripheral nervous system. J. Clin. Investig..

[57] Pitas R.E., Boyles J.K., Lee S.H., Foss D., Mahley R.W. (1987). Astrocytes synthesize apolipoprotein E and metabolize apolipoprotein E-containing lipoproteins. Biochim. Biophys. Acta.

[58] Mahley R.W. (1988). Apolipoprotein E: Cholesterol transport protein with expanding role in cell biology. Science.

[59] Namba Y., Tomonaga M., Kawasaki H., Otomo E., Ikeda K. (1991). Apolipoprotein E immunoreactivity in cerebral amyloid deposits and neurofibrillary tangles in Alzheimer’s disease and kuru plaque amyloid in Creutzfeldt-Jakob disease. Brain Res..

[60] Liu C.-C., Kanekiyo T., Xu H., Bu G. (2013). Apolipoprotein E and Alzheimer disease: Risk, mechanisms and therapy. Nat. Rev. Neurol..

[61] Dong L.M., Weisgraber K.H. (1996). Human apolipoprotein E4 domain interaction. Arginine 61 and glutamic acid 255 interact to direct the preference for very low density lipoproteins. J. Biol. Chem..

[62] Farrer L.A., Cupples L.A., Haines J.L., Hyman B., Kukull W.A., Mayeux R., Myers R.H., Pericak-Vance M.A., Risch N., van Duijn C.M. (1997). Effects of age, sex, and ethnicity on the association between apolipoprotein E genotype and Alzheimer disease. A meta-analysis. APOE and Alzheimer Disease Meta Analysis Consortium. JAMA.

[63] Strittmatter W.J., Saunders A.M., Schmechel D., Pericak-Vance M., Enghild J., Salvesen G.S., Roses A.D. (1993). Apolipoprotein E: High-avidity binding to beta-amyloid and increased frequency of type 4 allele in late-onset familial Alzheimer disease. Proc. Natl. Acad. Sci. USA.

[64] Yamazaki Y., Painter M.M., Bu G., Kanekiyo T. (2016). Apolipoprotein E as a Therapeutic Target in Alzheimer’s Disease: A Review of Basic Research and Clinical Evidence. CNS Drugs.

[65] Jonsson T., Stefansson H., Steinberg S., Jonsdottir I., Jonsson P.V., Snaedal J., Bjornsson S., Huttenlocher J., Levey A.I., Lah J.J. (2013). Variant of TREM2 associated with the risk of Alzheimer’s disease. N. Engl. J. Med..

[66] Colonna M., Wang Y. (2016). TREM2 variants: New keys to decipher Alzheimer disease pathogenesis. Nat. Rev. Neurosci..

[67] Filipello F., Morini R., Corradini I., Zerbi V., Canzi A., Michalski B., Erreni M., Markicevic M., Starvaggi-Cucuzza C., Otero K. (2018). The Microglial Innate Immune Receptor TREM2 Is Required for Synapse Elimination and Normal Brain Connectivity. Immunity.

[68] Colonna M. (2003). TREMs in the immune system and beyond. Nat. Rev. Immunol..

[69] Daws M.R., Sullam P.M., Niemi E.C., Chen T.T., Tchao N.K., Seaman W.E. (2003). Pattern recognition by TREM-2: Binding of anionic ligands. J. Immunol..

[70] Takahashi K., Rochford C.D.P., Neumann H. (2005). Clearance of apoptotic neurons without inflammation by microglial triggering receptor expressed on myeloid cells-2. J. Exp. Med..

[71] Turnbull I.R., Colonna M. (2007). Activating and inhibitory functions of DAP12. Nat. Rev. Immunol..

[72] Turnbull I.R., Gilfillan S., Cella M., Aoshi T., Miller M., Piccio L., Hernandez M., Colonna M. (2006). Cutting edge: TREM-2 attenuates macrophage activation. J. Immunol..

[73] Hao K., Di Narzo A.F., Ho L., Luo W., Li S., Chen R., Li T., Dubner L., Pasinetti G.M. (2015). Shared genetic etiology underlying Alzheimer’s disease and type 2 diabetes. Mol. Aspects Med..

[74] Annese A., Manzari C., Lionetti C., Picardi E., Horner D.S., Chiara M., Caratozzolo M.F., Tullo A., Fosso B., Pesole G. (2018). Whole transcriptome profiling of Late-Onset Alzheimer’s Disease patients provides insights into the molecular changes involved in the disease. Sci. Rep..

[75] Zhu X.-C., Tan L., Wang H.-F., Jiang T., Cao L., Wang C., Wang J., Tan C.-C., Meng X.-F., Yu J.-T. (2015). Rate of early onset Alzheimer’s disease: A systematic review and meta-analysis. Ann. Transl. Med..

[76] Gong C.-X., Liu F., Iqbal K. (2018). Multifactorial Hypothesis and Multi-Targets for Alzheimer’s Disease. J. Alzheimers Dis..

[77] Sanchez-Mut J.V., Gräff J. (2015). Epigenetic Alterations in Alzheimer’s Disease. Front Behav. Neurosci..

[78] Bradley-Whitman M.A., Lovell M.A. (2013). Epigenetic changes in the progression of Alzheimer’s disease. Mech. Ageing Dev..

[79] Ciceri F., Rotllant D., Maes T. (2017). Understanding Epigenetic Alterations in Alzheimer’s and Parkinson’s Disease: Towards Targeted Biomarkers and Therapies. Curr. Pharm. Des..

[80] Kouzarides T. (2007). Chromatin modifications and their function. Cell.

[81] Fischer A., Sananbenesi F., Wang X., Dobbin M., Tsai L.-H. (2007). Recovery of learning and memory is associated with chromatin remodelling. Nature.

[82] Zhang K., Schrag M., Crofton A., Trivedi R., Vinters H., Kirsch W. (2012). Targeted proteomics for quantification of histone acetylation in Alzheimer’s disease. Proteomics.

[83] Guan J.-S., Haggarty S.J., Giacometti E., Dannenberg J.-H., Joseph N., Gao J., Nieland T.J.F., Zhou Y., Wang X., Mazitschek R. (2009). HDAC2 negatively regulates memory formation and synaptic plasticity. Nature.

[84] Gräff J., Rei D., Guan J.-S., Wang W.-Y., Seo J., Hennig K.M., Nieland T.J.F., Fass D.M., Kao P.F., Kahn M. (2012). An epigenetic blockade of cognitive functions in the neurodegenerating brain. Nature.

[85] Yamakawa H., Cheng J., Penney J., Gao F., Rueda R., Wang J., Yamakawa S., Kritskiy O., Gjoneska E., Tsai L.-H. (2017). The Transcription Factor Sp3 Cooperates with HDAC2 to Regulate Synaptic Function and Plasticity in Neurons. Cell Rep..

[86] Wang C., Schroeder F.A., Wey H.-Y., Borra R., Wagner F.F., Reis S., Kim S.W., Holson E.B., Haggarty S.J., Hooker J.M. (2014). In vivo imaging of histone deacetylases (HDACs) in the central nervous system and major peripheral organs. J. Med. Chem..

[87] Wey H.-Y., Wang C., Schroeder F.A., Logan J., Price J.C., Hooker J.M. (2015). Kinetic Analysis and Quantification of [^11^C]Martinostat for in Vivo HDAC Imaging of the Brain. ACS Chem. Neurosci..

[88] Dobbin M.M., Madabhushi R., Pan L., Chen Y., Kim D., Gao J., Ahanonu B., Pao P.-C., Qiu Y., Zhao Y. (2013). SIRT1 collaborates with ATM and HDAC1 to maintain genomic stability in neurons. Nat. Neurosci..

[89] Bardai F.H., Price V., Zaayman M., Wang L., D’Mello S.R. (2012). Histone deacetylase-1 (HDAC1) is a molecular switch between neuronal survival and death. J. Biol. Chem..

[90] Gjoneska E., Pfenning A.R., Mathys H., Quon G., Kundaje A., Tsai L.-H., Kellis M. (2015). Conserved epigenomic signals in mice and humans reveal immune basis of Alzheimer’s disease. Nature.

[91] Xin S.-H., Tan L., Cao X., Yu J.-T., Tan L. (2018). Clearance of Amyloid Beta and Tau in Alzheimer’s Disease: From Mechanisms to Therapy. Neurotox Res..

[92] Jevtic S., Sengar A.S., Salter M.W., McLaurin J. (2017). The role of the immune system in Alzheimer disease: Etiology and treatment. Ageing Res. Rev..

[93] Mawuenyega K.G., Sigurdson W., Ovod V., Munsell L., Kasten T., Morris J.C., Yarasheski K.E., Bateman R.J. (2010). Decreased clearance of CNS beta-amyloid in Alzheimer’s disease. Science.

[94] Zhang B., Gaiteri C., Bodea L.-G., Wang Z., McElwee J., Podtelezhnikov A.A., Zhang C., Xie T., Tran L., Dobrin R. (2013). Integrated systems approach identifies genetic nodes and networks in late-onset Alzheimer’s disease. Cell.

[95] Kim-Ha J., Kim Y.-J. (2016). Age-related epigenetic regulation in the brain and its role in neuronal diseases. BMB Rep..

[96] Peleg S., Sananbenesi F., Zovoilis A., Burkhardt S., Bahari-Javan S., Agis-Balboa R.C., Cota P., Wittnam J.L., Gogol-Doering A., Opitz L. (2010). Altered histone acetylation is associated with age-dependent memory impairment in mice. Science.

[97] Tang B., Dean B., Thomas E.A. (2011). Disease- and age-related changes in histone acetylation at gene promoters in psychiatric disorders. Transl. Psychiatry.

[98] Nativio R., Donahue G., Berson A., Lan Y., Amlie-Wolf A., Tuzer F., Toledo J.B., Gosai S.J., Gregory B.D., Torres C. (2018). Dysregulation of the epigenetic landscape of normal aging in Alzheimer’s disease. Nat. Neurosci..

[99] Wang J., Yu J.-T., Tan M.-S., Jiang T., Tan L. (2013). Epigenetic mechanisms in Alzheimer’s disease: Implications for pathogenesis and therapy. Ageing Res. Rev..

[100] López-Otín C., Blasco M.A., Partridge L., Serrano M., Kroemer G. (2013). The hallmarks of aging. Cell.

[101] Brody H. (1955). Organization of the cerebral cortex. III. A study of aging in the human cerebral cortex. J. Comp. Neurol..

[102] Ball M.J. (1977). Neuronal loss, neurofibrillary tangles and granulovacuolar degeneration in the hippocampus with ageing and dementia. A quantitative study. Acta Neuropathol..

[103] Brizzee K.R., Ordy J.M., Bartus R.T. (1980). Localization of cellular changes within multimodal sensory regions in aged monkey brain: Possible implications for age-related cognitive loss. NBA.

[104] West M.J., Coleman P.D., Flood D.G., Troncoso J.C. (1994). Differences in the pattern of hippocampal neuronal loss in normal ageing and Alzheimer’s disease. Lancet.

[105] Gazzaley A.H., Thakker M.M., Hof P.R., Morrison J.H. (1997). Preserved number of entorhinal cortex layer II neurons in aged macaque monkeys. NBA.

[106] Rapp P.R., Gallagher M. (1996). Preserved neuron number in the hippocampus of aged rats with spatial learning deficits. Proc. Natl. Acad. Sci. USA.

[107] Woodruff-Pak D.S., Foy M.R., Akopian G.G., Lee K.H., Zach J., Nguyen K.P.T., Comalli D.M., Kennard J.A., Agelan A., Thompson R.F. (2010). Differential effects and rates of normal aging in cerebellum and hippocampus. PNAS.

[108] Rasmussen T., Schliemann T., Sørensen J.C., Zimmer J., West M.J. (1996). Memory impaired aged rats: No loss of principal hippocampal and subicular neurons. NBA.

[109] Burke S.N., Barnes C.A. (2006). Neural plasticity in the ageing brain. Nat. Rev. Neurosci..

[110] Dickstein D.L., Weaver C.M., Luebke J.I., Hof P.R. (2013). Dendritic spine changes associated with normal aging. Neuroscience.

[111] Peters A., Sethares C. (2002). The effects of age on the cells in layer 1 of primate cerebral cortex. Cereb. Cortex.

[112] Page T.L., Einstein M., Duan H., He Y., Flores T., Rolshud D., Erwin J.M., Wearne S.L., Morrison J.H., Hof P.R. (2002). Morphological alterations in neurons forming corticocortical projections in the neocortex of aged Patas monkeys. Neurosci. Lett..

[113] Dumitriu D., Hao J., Hara Y., Kaufmann J., Janssen W.G.M., Lou W., Rapp P.R., Morrison J.H. (2010). Selective changes in thin spine density and morphology in monkey prefrontal cortex correlate with aging-related cognitive impairment. J. Neurosci..

[114] Cesa R., Scelfo B., Strata P. (2007). Activity-dependent presynaptic and postsynaptic structural plasticity in the mature cerebellum. J. Neurosci..

[115] Tessier C.R., Broadie K. (2009). Activity-dependent modulation of neural circuit synaptic connectivity. Front Mol. Neurosci..

[116] Shinoda Y., Tanaka T., Tominaga-Yoshino K., Ogura A. (2010). Persistent synapse loss induced by repetitive LTD in developing rat hippocampal neurons. PLoS ONE.

[117] Lin Y.-T., Seo J., Gao F., Feldman H.M., Wen H.-L., Penney J., Cam H.P., Gjoneska E., Raja W.K., Cheng J. (2018). APOE4 Causes Widespread Molecular and Cellular Alterations Associated with Alzheimer’s Disease Phenotypes in Human iPSC-Derived Brain Cell Types. Neuron.

[118] Hong S., Beja-Glasser V.F., Nfonoyim B.M., Frouin A., Li S., Ramakrishnan S., Merry K.M., Shi Q., Rosenthal A., Barres B.A. (2016). Complement and microglia mediate early synapse loss in Alzheimer mouse models. Science.

[119] Roy D.S., Arons A., Mitchell T.I., Pignatelli M., Ryan T.J., Tonegawa S. (2016). Memory retrieval by activating engram cells in mouse models of early Alzheimer’s disease. Nature.

[120] Ridge P.G., Kauwe J.S.K. (2018). Mitochondria and Alzheimer’s Disease: The Role of Mitochondrial Genetic Variation. Curr. Genet. Med. Rep..

[121] Mosconi L., Rinne J.O., Tsui W.H., Berti V., Li Y., Wang H., Murray J., Scheinin N., Någren K., Williams S. (2010). Increased fibrillar amyloid-{beta} burden in normal individuals with a family history of late-onset Alzheimer’s. PNAS.

